# Association Between Heart Failure Etiology and All-Cause Mortality with Sex-Specific Considerations: Insights from the HEROES Registry

**DOI:** 10.3390/jcm15124759

**Published:** 2026-06-18

**Authors:** Michał Tarnowski, Robert Morawiec, Agata Galas, Agata Tymińska, Katarzyna Byczkowska, Jarosław Kasprzak, Aleksander Siniarski, Anna Żarek-Starzewska, Agnieszka Major, Adrian Stefański, Małgorzata Zachura, Jarosław Drożdż, Iwona Gorczyca-Głowacka

**Affiliations:** 1 Department of Cardiology, Holy Spirit Specialist Hospital, 27-600 Sandomierz, Poland; michal.tarnowski8@gmail.com; 2 Department of Emergency Medicine and Disaster Medicine, Medical University of Lodz, 90-549 Lodz, Poland; robert.morawiec@umed.lodz.pl; 3 Department of Cardiology and Internal Diseases, Military Institute of Medicine, 04-141 Warsaw, Poland; 4 First Department of Cardiology, Medical University of Warsaw, 02-097 Warsaw, Poland; 5 Heart Failure and Transplantology Department, National Institute of Cardiology—National Research Institute, 05-400 Warsaw, Poland; 6 1st Department and Chair of Cardiology, Medical University of Lodz, 90-425 Lodz, Poland; kasprzak@ptkardio.pl; 7 Department of Coronary Artery Disease and Heart Failure, St. John Paul II Hospital, 31-202 Krakow, Poland; 8 Department of Coronary Artery Disease and Heart Failure, Institute of Cardiology, Faculty of Medicine, Jagiellonian University Medical College, 31-202 Krakow, Poland; 9 Department of Cardiology, Hypertension and Internal Medicine, Medical University of Warsaw, 02-907 Warsaw, Poland; 10 1st Clinic of Cardiology and Electrotherapy, Swietokrzyskie Cardiology Center, 25-736 Kielce, Poland; 11 Department of Hypertension and Diabetology, Faculty of Medicine, Medical University of Gdansk, 80-214 Gdansk, Poland; adrian.stefanski@gumed.edu.pl; 12 Collegium Medicum, Jan Kochanowski University, 25-516 Kielce, Poland; 13 2nd Department of Cardiology, Medical University of Lodz, 90-549 Lodz, Poland; jaroslaw.drozdz@umed.lodz.pl

**Keywords:** etiology, heart failure, ischemic, prognosis

## Abstract

**Background:** Heart failure (HF) is a complex clinical syndrome, and its prognosis depends on many factors, including its etiology and the patient’s sex. We aimed to perform gendered evaluations on ischemic etiology’s impact on HF prognosis. **Methods:** Hospitalized patients and outpatients were enrolled in the Heart Failure Observational Study (HEROES), which is a prospective, multicenter cohort study, between April 2022 and January 2024. The primary endpoint was all-cause mortality. **Results:** Among 1410 patients included in the analysis (28.4% females and 71.6% males), 41.1% had ischemic HF etiology, and 58.9% had non-ischemic HF etiology. Ischemic etiology was identified in 28.5% of females and 46.0% of males; *p* < 0.001. The adjusted hazard ratio (aHR) was 1.16 (95% CI 0.85–1.58; *p* = 0.363) for all-cause mortality in the non-ischemic group relative to the ischemic reference category. The aHR for all-cause mortality in women relative to men was 1.14 (95% CI: 0.67–1.94; *p* = 0.633) for ischemic HF and 0.85 (95% CI: 0.56–1.27; *p* = 0.420) for non-ischemic HF. **Conclusions:** We found that ischemic and non-ischemic etiologies are associated with comparable all-cause mortality risk in patients with HF. Sex-stratified analyses revealed no significant mortality differentials between women and men within either etiologic category.

## 1. Introduction

The prevalence of heart failure (HF) constitutes a global pandemic, with an estimated prevalence of 1% to 3% in different populations. The prevalence of HF is notably influenced by various factors, including age, lifestyle, and pre-existing health conditions [1,2,3]. As the aging population continues to increase in number, coupled with an increasing prevalence of cardiovascular risk factors, the burden of HF is expected to worsen substantially [4]. Thus, robust exploration of etiologic factors contributing to HF is imperative for the development of effective prevention and management strategies. Whether ischemic etiology confers an independent prognostic disadvantage relative to non-ischemic etiologies remains a subject of ongoing investigation, with certain studies reporting inferior outcomes in selected HF populations [5].

The etiologic profile, disease trajectory, and prognosis of HF differ appreciably between women and men [6], largely owing to sex-dependent disparities in the prevalence of ischemic heart disease, valvular pathology, and metabolic comorbidities. The HF population is inherently heterogeneous, with incidence patterns varying across age strata; advances in the early detection and treatment of coronary artery disease, hypertension, and valvular heart disease have reshaped the contemporary sex–etiology landscape relative to prior decades [7,8,9].

Given the evolving epidemiological profile of HF and the persistent uncertainty regarding whether etiologic classification carries independent prognostic weight after rigorous confounding adjustment, the present investigation was conceived to address this gap using contemporary data from a nationwide registry, with particular attention to potential sex-based heterogeneity in the etiology–mortality relationship.

## 2. Materials and Methods

### 2.1. Study Population

The present investigation constituted a secondary analysis of the Heart Failure Observational Study (HEROES), a multicenter prospective registry [10]. The primary objective was to evaluate the association between HF etiology (ischemic versus non-ischemic) and all-cause mortality, employing propensity score weighting to mitigate confounding bias. Secondary objectives were to characterize sex-specific demographic, clinical, and pharmacotherapeutic profiles within each etiologic category and to compare all-cause mortality between women and men within the ischemic and non-ischemic strata separately.

Both inpatients and outpatients with HF were included in the study. The diagnosis of HF was made by the researchers based on current European Society of Cardiology guidelines, taking into consideration symptoms, signs, and abnormalities found on echocardiographic and/or laboratory testing.

The primary exposure was etiologic classification, operationalized as a binary variable (ischemic versus non-ischemic). The primary endpoint was all-cause mortality treated as a time-to-event outcome, with follow-up measured in months from the index enrollment date. Patients alive at the end of the observation period or lost to follow-up were censored at their last known contact date. The target estimand was the average treatment effect (ATE) in the population, estimated via inverse probability of treatment weighting. A comprehensive summary of the study design elements—including exposure definitions, outcome specifications, covariate domains, and statistical estimands—is provided in Table S1. The study design, enrollment-to-analytical-stratification patient flow, and sequential methodological framework are depicted in Figure S1.

Two major groups were identified according to HF etiology: ischemic and non-ischemic. When numerous etiologic factors were observed, the predominant factor was considered the primary cause. Patients were classified into a particular etiologic group at the time of enrollment in the study, rather than during data analysis.

The criteria for assignment to a specific etiologic group included the following:

- Ischemic etiology—patients with a history of prior myocardial infarction or coronary intervention, either coronary artery bypass graft surgery or percutaneous coronary intervention;
- Non-ischemic etiology—patients with hypertensive, valvular, arrhythmic, and other HF etiologies.

### 2.2. Covariates

Comprehensive baseline data on medical history, demographics, pharmacotherapy, comorbidities, and diagnostic test results were obtained.

Following the contemporary HF classification, patients were stratified into three phenotypes based on the left ventricular ejection fraction (LVEF). In particular, HFrEF was determined by an LVEF below 40%, while the HFmrEF and HFpEF categories included participants with LVEF values of 40–49% and ≥50%, respectively.

The estimated glomerular filtration rate (eGFR), calculated using the Modification of Diet in Renal Disease (MDRD) equation, was used to assess patients’ kidney function.

### 2.3. Follow-Up

Data on all-cause mortality during the follow-up period were obtained from the Polish Ministry of Digital Affairs in accordance with the General Data Protection Regulation (GDPR). The dataset included only the date of death from any cause. This allowed for the collection of complete clinical and survival data for all 1422 participants of the HEROES study, with no patients lost to follow-up. No follow-up visits were scheduled according to protocol, and all-cause mortality was the only outcome assessed.

### 2.4. Ethical Issues

The Bioethical Committee at the Medical University of Lodz approved the implementation of the Heart Failure Observational Study of the Polish Cardiac Society (No. RNN/316/20/KE with the update KE/762/23). Informed consent was obtained from all the study participants.

### 2.5. Statistical Analysis

All hypothesis tests were conducted at a two-sided significance level of α = 0.05, with *p* < 0.05 denoting statistical significance. No formal multiplicity adjustment was applied to the primary analysis, given the single primary comparison (ischemic versus non-ischemic); exploratory subgroup analyses were interpreted with appropriate circumspection.

Continuous variables are summarized as medians (interquartile range) and compared with the Wilcoxon rank-sum test; categorical variables are expressed as frequencies (%) and compared with Pearson’s χ^2^ test. Confidence interval methods and additional specifications are reported in the footnotes of Table 1 and Table 2.

Confounding between heart failure etiology and mortality was addressed by propensity score weighting (PSW) via entropy balancing, targeting the average treatment effect (ATE).

Thirteen prognostically relevant baseline covariates were entered into the weighting model (complete list and rationale in Table S1). Post-exposure pharmacotherapy and variables constitutive of the ischemic phenotype were excluded a priori to avoid overadjustment bias.

Balance was verified via standardized mean differences against a pre-specified |SMD| < 0.10 threshold (Tables S2 and S3, Figure S2).

**Figure 1:**
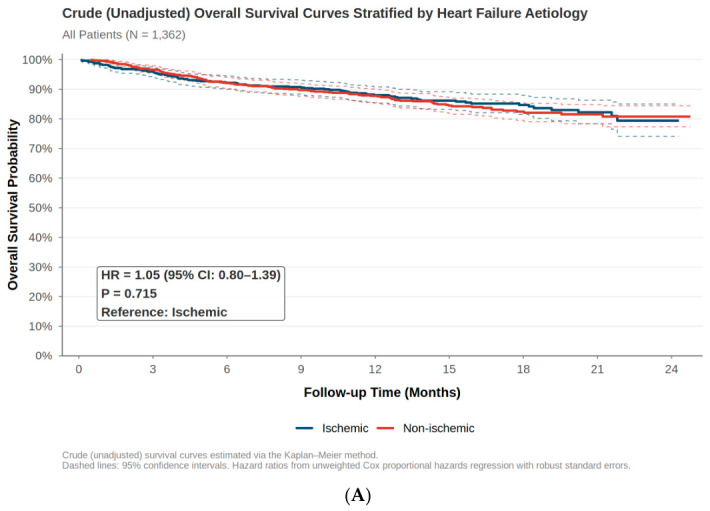

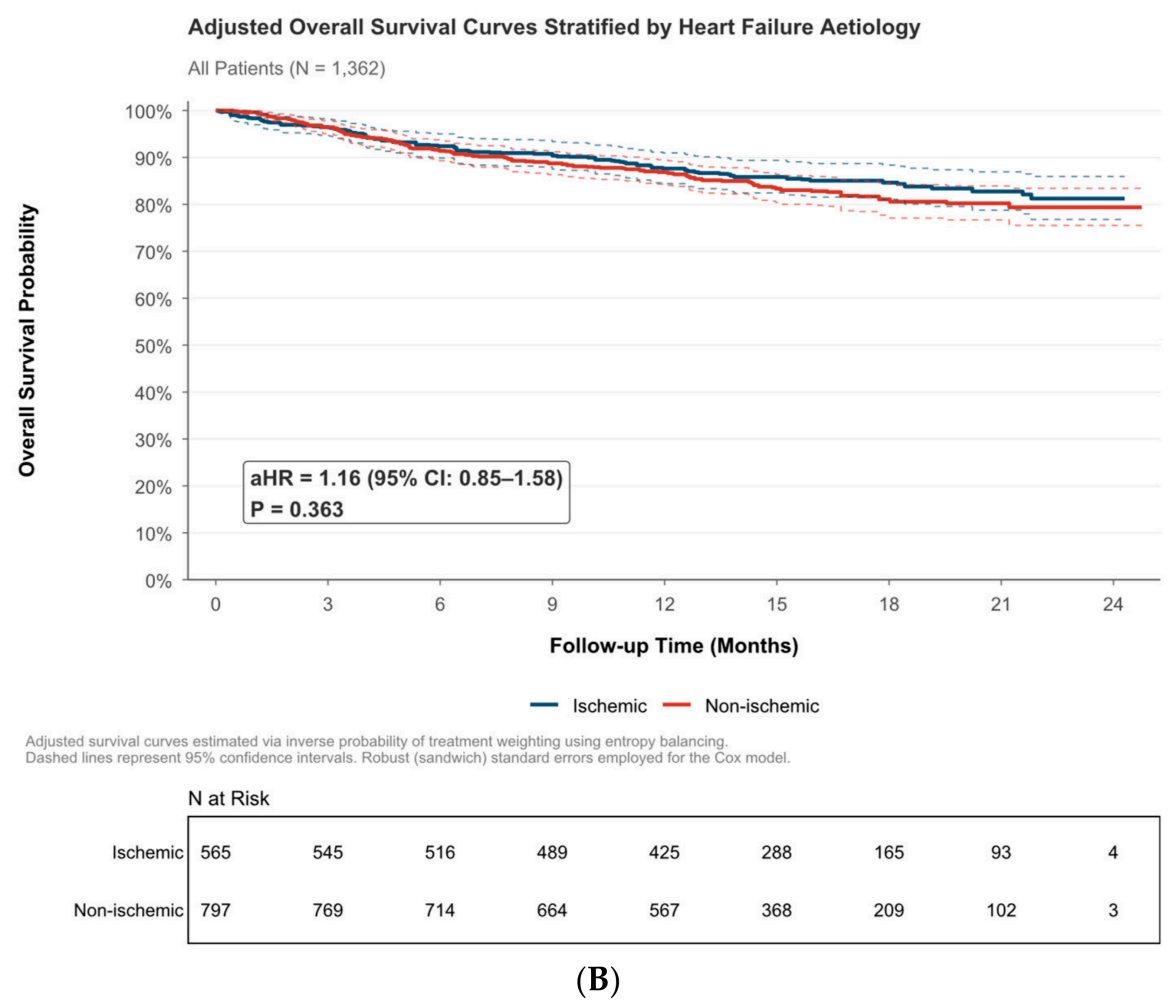
(**A**) Crude (unadjusted) overall survival curves for all-cause mortality stratified by heart failure etiology (ischemic vs. non-ischemic) in the propensity-score-eligible analytical cohort (N = 1362), estimated via the Kaplan–Meier method without propensity-score weighting. Dashed lines represent 95% confidence intervals; numbers at risk are displayed beneath the plot. Hazard ratio derived from unweighted Cox proportional hazards regression with robust standard errors. (**B**) Adjusted overall survival curves for all-cause mortality stratified by heart failure etiology (ischemic vs. non-ischemic) in the full analytical cohort (N = 1362), estimated using propensity score-weighted Kaplan–Meier method with entropy balancing. Dashed lines represent 95% confidence intervals; numbers at risk are displayed beneath the plot. Adjusted hazard ratio derived from weighted Cox proportional hazards regression with robust standard errors.

**Figure 2:**
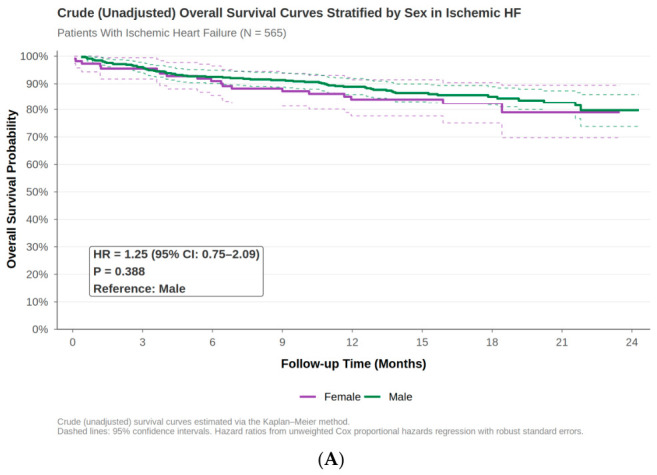

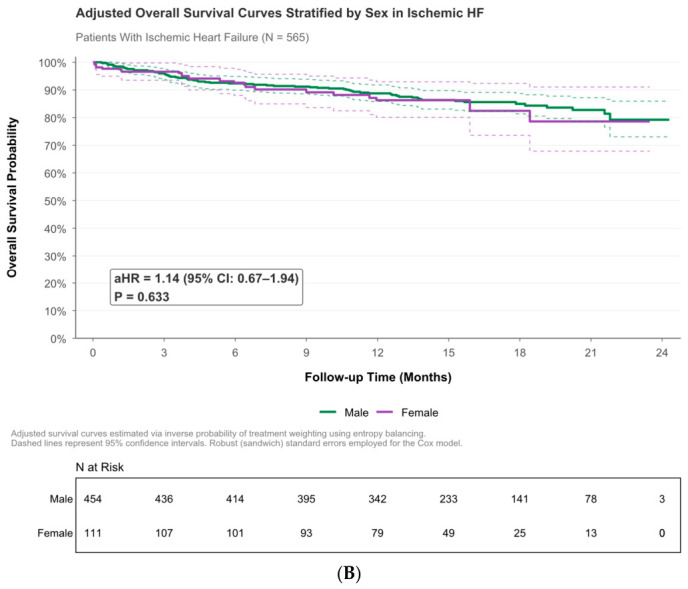
(**A**) Crude (unadjusted) overall survival curves for all-cause mortality stratified by sex (male vs. female) within the ischemic heart failure subgroup (N = 565). Dashed lines represent 95% confidence intervals; robust standard errors were employed. (**B**) Adjusted overall survival curves for all-cause mortality stratified by sex (male vs. female) within the ischemic heart failure subgroup (n = 565), with propensity score weights re-estimated via entropy balancing on 12 covariates. Dashed lines: 95% CI; robust standard errors.

**Figure 3:**
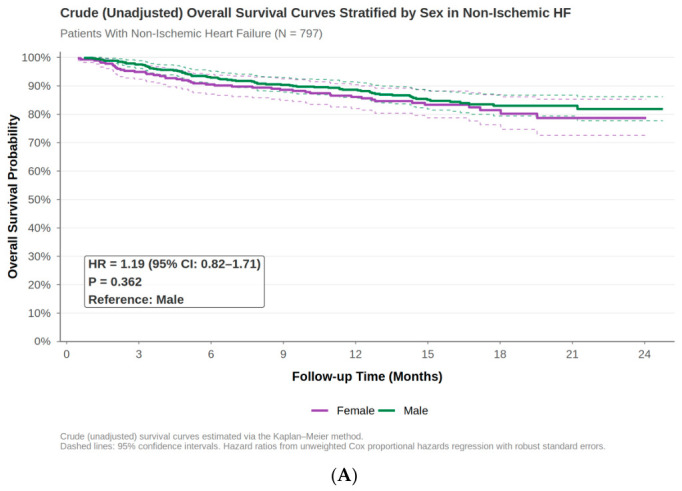

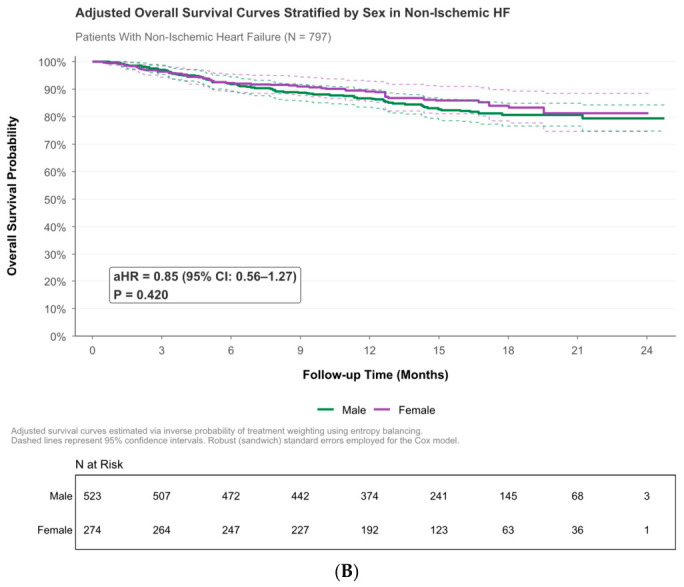
(**A**) Crude (unadjusted) overall survival curves for all-cause mortality stratified by sex (male vs. female) within the non-ischemic heart failure subgroup (N = 797). Dashed lines represent 95% confidence intervals; robust standard errors were employed. (**B**) Adjusted overall survival curves for all-cause mortality stratified by sex (male vs. female) within the non-ischemic heart failure subgroup (n = 797), with propensity score weights re-estimated via entropy balancing on 12 covariates. Dashed lines: 95% CI; robust standard errors.

Adjusted survival curves were estimated using the weighted Kaplan–Meier (KM) method, incorporating PSW weights from entropy balancing, and they were visualized with 95% confidence interval bands. Three principal survival analyses were conducted: ischemic versus non-ischemic etiology in the full cohort (Figure 1); women versus men within the ischemic subgroup, with PSW re-estimated within the stratum (Figure 2); and women versus men within the non-ischemic subgroup (Figure 3). Effect sizes were derived from weighted Cox proportional hazards models with robust (sandwich) standard errors. Results are expressed as adjusted hazard ratios (aHRs) with 95% CIs and Wald-test *p*-values. A doubly robust specification—incorporating both PSW weights and direct covariate adjustment for age, sex, NYHA class, diabetes mellitus, and chronic kidney disease—was fitted as a secondary model to provide additional protection against residual confounding and model misspecification (Table S4, Model 4).

The proportional hazards (PH) assumption was assessed using the Schoenfeld residuals test with both global and covariate-specific *p*-values (Figure S3). The discriminative capacity of the Cox models was quantified using Harrell’s concordance index, computed with bootstrap internal validation (B = 500 resamples).

Robustness was evaluated through alternative weighting specifications, E-value computation for unmeasured confounding, pre-specified subgroup interaction tests, and influence diagnostics (Table S5).

## 3. Results

### 3.1. Study Population and Baseline Characteristics

The analytical cohort comprised 1410 patients, with a median age of 69.3 (IQR: 60.3–76.2) years, including 400 females (28.4%) and 1010 males (71.6%).

Concerning the primary etiology of HF, 579 (41.1%) were classified as having ischemic HF etiology, whereas 831 (58.9%) constituted the non-ischemic group, encompassing dilated cardiomyopathy (n = 191, 13.5%), hypertensive HF (n = 170, 12.1%), valvular HF (n = 151, 10.7%), and other etiologies (n = 319, 22.6%).

Ischemic etiology was identified in 114 women (28.5% of all females) and 465 men (46.0% of all males; *p* < 0.001), confirming the well-established male predominance in ischemic heart disease.

The predominance of hospitalized patients was evident, with 1131 individuals (80.2%) enrolled during an inpatient admission and 279 (19.8%) recruited in outpatient settings. Regarding the LVEF-based phenotypic classification, available in 1210 patients (85.8%), the distribution revealed 627 patients (51.8%) with HFrEF, 262 (21.7%) with HFmrEF, and 321 (26.5%) with HFpEF. The 200 patients (14.2%) lacking LVEF classification were retained in the primary analysis and addressed separately in the sensitivity analysis program (Table S5).

### 3.2. Baseline Characteristics and Between-Group Imbalances

Pervasive and clinically meaningful imbalances between ischemic and non-ischemic groups were observed across demographic, hemodynamic, comorbidity, and pharmacotherapeutic domains (Table 1), with 13 of 15 covariates reaching statistical significance and underscoring the necessity of rigorous confounding adjustment (Tables S2 and S3; Figure S2).

The sex-stratified characterization within each etiologic group (Table 2) further demonstrated that women in both strata were older and exhibited higher rates of HFpEF, lower Kansas City Cardiomyopathy Questionnaire-12 (KCCQ-12) scores, and distinct pharmacotherapeutic profiles.

### 3.3. Propensity Score Weighting and Covariate Balance

Entropy balancing eliminated the substantial pre-weighting imbalances and achieved complete first-moment balance across all 13 covariates, with effective sample sizes of 435.7 (77.1% retention) and 705.1 (88.5% retention) in the ischemic and non-ischemic strata, respectively (Tables S2 and S3, Figure S2).

### 3.4. Survival Analysis: Ischemic vs. Non-Ischemic Etiology

Over a median follow-up of 14.8 months (IQR: 11.5–18.4), a total of 207 all-cause deaths occurred in the analytical cohort available for the weighted survival analysis (N = 1362 after exclusion of 48 observations with incomplete covariate data). Crude mortality rates were virtually identical between the two etiologic groups: 90 deaths among ischemic patients (15.5%) and 129 among non-ischemic patients (15.5%; *p* > 0.999).

Crude (unadjusted) Kaplan–Meier curves for the primary etiology comparison are presented in Figure 1A, demonstrating closely superimposed survival trajectories with HR = 1.05 (95% CI: 0.80–1.39; *p* = 0.715) for non-ischemic relative to ischemic patients.

The propensity score-weighted Kaplan–Meier survival curves for the primary comparison are presented in Figure 1B.

Both curves commenced at unity and maintained closely overlapping trajectories through the initial six months of follow-up, with 12-month weighted survival estimates of approximately 91% in the ischemic group and 89% in the non-ischemic group. A modest and gradual separation of the curves became apparent beyond the 12-month landmark; however, 95% CI bands overlapped extensively throughout the entire observation window.

The primary weighted Cox proportional hazards model yielded an adjusted hazard ratio of 1.16 (95% CI: 0.85–1.58; *p* = 0.363) for all-cause mortality in the non-ischemic group relative to the ischemic reference category (Table S4, Model 1).

A doubly robust specification incorporating both entropy balancing weights and direct covariate adjustment for age, sex, NYHA functional class, diabetes mellitus, and chronic kidney disease yielded an adjusted hazard ratio of 1.12 (95% CI: 0.82–1.54; *p* = 0.469; Table S4, Model 4).

The proportional hazards assumption was satisfied (global Grambsch–Therneau test: *p* = 0.457; Figure S3).

Clinically, these effect estimates indicate that ischemic and non-ischemic heart failure etiologies confer comparable risk of all-cause death during medium-term follow-up in this contemporary registry.

### 3.5. Sex-Stratified Survival Analysis

Sex-stratified analyses were performed within each etiologic subgroup, with propensity score weights re-estimated on twelve baseline covariates (ischemic: n = 565, 84 events; non-ischemic: n = 797, 123 events). All within-stratum covariates achieved balance (Figure S2).

Crude (unadjusted) Kaplan–Meier curves stratified by sex within each aetiologic subgroup are presented in Figure 2A and Figure 3A. The crude hazard ratio for female versus male mortality was 1.25 (95% CI: 0.75–2.09; *p* = 0.388) within the ischemic subgroup and 1.19 (95% CI: 0.82–1.71; *p* = 0.362) within the non-ischemic subgroup, demonstrating that no marked unadjusted sex-related survival differential existed in either stratum prior to propensity-score weighting.

Within the ischemic HF subgroup, the crude mortality rate was numerically higher among women (21 out of 114; 18.4%) than among men (69 out of 465; 14.8%), although this unadjusted disparity did not withstand confounder adjustment. The weighted Kaplan–Meier curves (Figure 2B) exhibited broadly overlapping trajectories with extensive confidence interval superimposition throughout the observation period.

The weighted Cox model yielded an adjusted hazard ratio of 1.14 (95% CI: 0.67–1.94; *p* = 0.633) for women relative to men (Table S4, Model 2).

Within the non-ischemic HF subgroup, women constituted a substantially larger proportion of the non-ischemic cohort (286 out of 831; 34.4%) compared with the ischemic group (114 out of 579; 19.7%). Crude mortality rates were 14.9% in men and 16.8% in women. The weighted Kaplan–Meier curves (Figure 3B) demonstrated a slight survival advantage for women in the early follow-up period that attenuated beyond the 12-month landmark, with overlapping confidence bands throughout.

The adjusted hazard ratio was 0.85 (95% CI: 0.56–1.27; *p* = 0.420; Table S4, Model 3), indicating a non-significant 15% reduction in mortality risk for women relative to men.

Both sex-stratified analyses were performed under constrained statistical power (ischemic women: n = 114, 21 events; non-ischemic women: n = 286, 48 events), and the absence of statistically significant sex–mortality associations within either stratum should be interpreted as inconclusive rather than confirmatory of the null.

### 3.6. Model Validation

Bootstrap-corrected C-indices were 0.494 for the etiology-only model (as expected for a single binary exposure designed for causal rather than predictive inference) and 0.673 for the doubly robust specification.

### 3.7. Sensitivity Analyses

The primary effect estimate proved stable across four alternative weighting specifications and an extended 14-covariate model including HF phenotype (aHR range: 1.05–1.17; all *p* > 0.35; Table S5). The E-value of 1.58 quantifies the minimum confounding strength required to nullify the observed estimate. No statistically significant effect modification emerged across pre-specified subgroups (Figure S4), and influence diagnostics revealed no aberrant observations.

## 4. Discussion

The multicenter HEROES registry, encompassing a nationwide cohort of 1410 patients across 41 Polish cardiology centers, provides contemporary real-world evidence regarding the prognostic implications of ischemic versus non-ischemic HF etiology following rigorous confounding adjustment via propensity score weighting.

The principal findings of this investigation may be summarized as follows: First, non-ischemic etiology predominated in both sexes, yet ischemic HF was significantly more prevalent among men. Second, ischemic and non-ischemic HF etiologies were associated with comparable all-cause mortality risk. Third, sex-stratified analyses revealed no significant mortality differentials between women and men within either etiologic category.

The classification of HF etiologies in our study was based on documentation within the HEROES registry, which may vary in consistency across healthcare centers. The broad categorization of etiologies may oversimplify the heterogeneity within each category, potentially obscuring more nuanced associations. The etiologic classification of HF into ischemic and non-ischemic is relatively general; however, it remains in use due to its clinical utility [11].

Concerning the primary etiology of HF, 41% were classified as having ischemic HF etiology. These HF results resemble those from other studies. In the Italian population, the proportion of patients with ischemic HF was similar to the results of this study and amounted to 44% [12]. Comparably, Kozman et al. [13] showed that in SwedeHF (Swedish Heart Failure Registry), concerning the primary etiology of HF, 38% of patients had ischemic HF. Furthermore, this research highlights an evolving temporal pattern in HF causes. Longitudinal data analysis revealed significant changes, specifically among HFrEF patients, where the initial prevalence of ischemic factors was eventually surpassed by ‘other’ etiologies as the study concluded. This transition is interpreted by the authors as a sign of successful interventions in ischemic heart disease prevention, echoing findings from the prior assessment [13]. In the study including patients with HF hospitalized between 2014 and 2019, there was a significant decrease in the percentage of cases with ischemic etiology over successive time periods for the whole HF population [14].

As expected, our study showed that non-ischemic etiology predominated in both sexes, yet ischemic HF was significantly more prevalent among men (46% vs. 29%). Similar findings have been reported in the Global Congestive Heart Failure (G-CHF) registry, where ischemic etiology predominated in men (46% vs. 27%). Moreover, fewer women than men had an LVEF ≤ 40% (52% vs. 66%) [15]. Notably, consistent results were observed in a large cohort study including 499,153 individuals, in which more than twice as many men as women had LVEF < 50%. This distribution is likely explained by underlying pathophysiological mechanisms and risk factor profiles associated with coronary artery disease, which is more prevalent in men than in women [16]. As is well known, cardiovascular risk factors differ between men and women, which may shape the etiology of HF. In our study, as expected, ischemic etiology was more common in men than in women. Men were more likely to be past or current smokers, whereas women were older.

The studies published to date do not provide a clear answer as to whether there is significant prognostic significance regarding the etiology. In the presented study, the primary analysis demonstrates that, following rigorous adjustment for 13 confounders via entropy balancing, ischemic and non-ischemic HF etiologies were associated with comparable all-cause mortality risk over a median follow-up approaching 15 months. The consistency between the single-predictor weighted model (aHR = 1.16) and the doubly robust specification (aHR = 1.12) strengthens confidence in the robustness of this null finding, which is further corroborated by the comprehensive sensitivity analysis program.

Evidence suggests that an ischemic origin in HF often correlates with the poorest outcomes. Kozman et al. [13] identified ischemia as the primary driver of mortality and hospitalization due to HF, though this was limited to the HFrEF patients. This is supported by further research indicating that while ischemic etiology negatively affects most outcomes across all HF phenotypes, its impact is most acute regarding HFrEF and recurrent ischemic incidents [17]. In a group of real-world outpatients with HF from various causes, those with ischemic heart disease faced the greatest adjusted risk of cardiovascular mortality relative to patients with other HF causes [18]. However, our findings indicate that the Italian study failed to confirm the impact of ischemic etiology on patient outcomes [12]. Rywik et al. [19] demonstrated that ischemic etiology did not serve as a significant prognostic factor across any HF phenotypes. It should be emphasized that the differences in the study results referring to prognosis in HF patients depending on etiology may stem from variations between the study cohorts, but even more importantly, from differing definitions of HF etiology. When the various classification schemes were compared, a modified number-of-diseased-vessels classification, in which patients with single-vessel disease and no prior history of revascularization or myocardial infarction were classified as non-ischemic, provided the most prognostic power. A definition of ischemic cardiomyopathy that incorporated this definition had more prognostic power than the traditional definition [20].

Moreover, in our study, sex-stratified analyses revealed no significant mortality differentials between women and men within either etiologic category, although limited statistical power in the female subgroups constrains the interpretive reach of these null findings. In some previous studies, women have shown better survival rates than men [21,22,23], a finding that is more pronounced in patients with non-ischemic HF [24,25].

The meta-analysis, which included 8791 men and 2851 women randomized in five clinical trials, showed that the 1-year Kaplan–Meier survival estimates varied by gender and etiology (female non-ischemic group, HR 0.88 [95% CI 0.85 to 0.89]; female ischemic group, HR 0.83 [95% CI 0.81 to 0.85]; male non-ischemic group, HR 0.84 [95% CI 0.83 to 0.85]; male ischemic group, HR 0.79 [95% CI 0.78 to 0.81]). After adjustment, female gender (HR 0.77 [95% CI 0.69 to 0.85]) and non-ischemic HF etiology (HR 0.80 [95% CI 0.72 to 0.89]) were associated with longer survival time [26]. The MAGGIC meta-analysis of 31 studies showed that survival was better for women with HF compared with men, irrespective of HF subtype. This survival benefit was slightly more marked in non-ischemic HF but is attenuated by concomitant diabetes [27].

Our study has several implications for clinical practice. As is well known, women are underrepresented in clinical trials, and patients in clinical trials are very deliberately selected. Our sample, used in a real-life study, is more representative than clinical randomization trials. On the other hand, our findings may differ from those of large registries due to the limited size of certain subgroups, particularly women with ischemic etiology. Women with HF have been undertreated compared to men. This difference has been attributed to women’s more unfavorable clinical profile, often entailing older age and common comorbidities [28]. However, we found significant differences in the prescription of medical treatment recommended in HF in ischemic and non-ischemic groups. Taken together, these data, along with our clinical experience, underscore the need for further studies addressing prognosis in relation to both HF etiology and patient sex.

## 5. Limitations

Our research has certain limitations that should be acknowledged. These mainly arise from the registry-based nature of the data used. First, some patients had missing data. Second, the collected information was restricted to what investigators put into the standardized case report form. Third, numerous issues relate to HF etiology. The broad classification of etiologies could oversimplify the diversity within each group, possibly masking more precise associations. Fourth, pharmacotherapy was deliberately excluded from propensity score weighting and outcome adjustment because medications constitute post-exposure variables on the causal pathway between heart failure etiology and mortality; their adjustment would induce overadjustment bias. The HEROES registry, moreover, does not contain longitudinal medication data with which to model time-varying treatment patterns via marginal structural models, the only methodologically appropriate framework for such an analysis. Residual confounding by medication patterns, although quantitatively bounded by the sensitivity analyses reported in Table S5, cannot be formally excluded. Lastly, the observational nature of our study precludes the establishment of causal relationships between HF etiologies and all-cause mortality. The sex-stratified analyses were underpowered, particularly among ischemic women (n = 114; 21 events), precluding definitive conclusions regarding sex-based heterogeneity in the etiology–mortality relationship; larger cohorts with event-enriched female subgroups would be required to resolve this question. In contrast to the mentioned points, it is important to underline that the HEROES population mirrors a typical cohort of Polish patients with HF. This study offers strong representativeness in its findings, capturing real-world challenges in HF patient care and enabling identification of risk factors deserving special focus in tailored, etiology-specific management approaches to improve outcomes for patients with HF.

Over the last few decades, the prognosis of patients with HF has improved remarkably. In addition to medical treatment, a large body of evidence shows that a variety of clinical factors may significantly influence the etiology of HF.

## 6. Conclusions

The present investigation, drawing upon the multicenter HEROES registry encompassing 1410 patients with HF, demonstrates that ischemic and non-ischemic etiologies are associated with comparable all-cause mortality risk. Sex-stratified analyses revealed no significant mortality differentials between women and men within either etiologic category. These findings carry implications for both clinical practice and future research. The absence of an overall mortality differential between ischemic and non-ischemic etiologies and sex indicates that etiologic classification alone should not be accorded independent prognostic weight in risk stratification algorithms for the general HF population.

## Figures and Tables

**Table 1:** Baseline demographic, clinical, and pharmacotherapeutic characteristics of patients with heart failure stratified by etiological classification (ischemic vs. non-ischemic), HEROES registry.

Characteristic	N	Overall N = 1410	95% CI	Ischemic N = 579	95% CI	Non-Ischemic N = 831	95% CI	*p*
***Demographics***								
Age, years	1410	69.3 (60.3–76.2)	67.6–69.0	70.1 (64.0–76.3)	69.2–70.8	68.3(56.8–76.1)	65.6–67.8	<0.001
Sex, male	1410	1010 (71.6%)	69.2–73.9	465 (80.3%)	76.9–83.4	545 (65.6%)	62.3–68.8	<0.001
***Anthropometrics and vital signs***								
Body mass index, kg/m^2^	1404	27.9 (24.8–32.1)	28.2–28.8	27.7 (24.8–31.4)	27.7–28.5	28.2(24.9–32.4)	28.4–29.2	0.045
Heart rate, bpm	1370	77 (67–90)	78–80	75 (65–85)	74–77	80 (70–92)	80–83	<0.001
Systolic BP, mmHg	1370	127 (113–140)	126–129	127 (114–140)	125–129	128 (113–142)	127–130	0.355
Diastolic BP, mmHg	1370	78 (70–85)	77–78	77 (70–83)	75–78	80 (70–86)	78–80	0.003
***Heart failure classification***								
HFrEF	1210	627 (51.8%)	49.0–54.6	305 (61.2%)	56.9–65.4	322 (45.2%)	41.6–48.9	<0.001
HFmrEF		262 (21.7%)	19.4–24.0	118 (23.7%)	20.1–27.6	144 (20.2%)	17.4–23.3	
HFpEF		321 (26.5%)	24.1–29.1	75 (15.1%)	12.1–18.4	246 (34.6%)	31.1–38.1	
***Disease history***								
HF duration, years	1410	2.9 (0.9–8.8)	4.3–5.1	4.5 (1.2–11.5)	5.6–7.1	2.2 (0.8–6.9)	3.3–4.1	<0.001
<1 year		387 (27.4%)	25.2–29.8	112 (19.3%)	16.3–22.7	275 (33.1%)	30.0–36.3	<0.001
1–3 years		321 (22.8%)	20.6–25.0	133 (23.0%)	19.7–26.5	188 (22.6%)	19.9–25.6	
>3 years		702 (49.8%)	47.2–52.4	334 (57.7%)	53.6–61.7	368 (44.3%)	40.9–47.7	
Visit: hospitalization	1410	1131 (80.2%)	78.1–82.2	446 (77.0%)	73.5–80.3	685 (82.4%)	79.7–84.9	0.015
***Functional status***								
NYHA I–II	1370	689 (50.3%)	47.6–52.9	319 (56.2%)	52.1–60.2	370 (46.1%)	42.7–49.6	<0.001
NYHA III–IV		681 (49.7%)	47.1–52.4	249 (43.8%)	39.8–47.9	432 (53.9%)	50.4–57.3	
***Renal and heart function***								
eGFR, mL/min/1.73 m^2^	1204	60.3 (44.8–77.4)	59.5–62.2	58.7 (44.7–75.2)	57.6–61.8	61.2 (44.8–78.1)	59.9–63.5	0.169
LVEF, %	1210	38.0 (27.0–50.0)	37.5–39.0	35.0 (26.0–44.8)	34.5–36.5	40.0 (27.8–55.0)	39.0–41.5	<0.001
***Comorbidities***								
Arterial hypertension	1398	953 (68.2%)	65.7–70.6	421 (73.2%)	69.5–76.7	532 (64.6%)	61.3–67.9	<0.001
Atrial fibrillation	1398	723 (51.7%)	49.1–54.3	255 (44.3%)	40.3–48.4	468 (56.9%)	53.5–60.2	<0.001
Diabetes mellitus	1398	532 (38.1%)	35.5–40.6	281 (48.9%)	44.8–53.0	251 (30.5%)	27.4–33.7	<0.001
Stroke/TIA	1410	128 (9.1%)	7.7–10.7	55 (9.5%)	7.3–12.1	73 (8.8%)	7.0–10.9	0.715
Myocardial infarction	1398	482 (34.5%)	32.0–37.0	423 (73.6%)	69.8–77.0	59 (7.2%)	5.6–9.1	<0.001
Stable angina	1398	430 (30.8%)	28.4–33.2	300 (52.2%)	48.1–56.2	130 (15.8%)	13.4–18.4	<0.001
Chronic kidney disease	1398	378 (27.0%)	24.8–29.4	185 (32.2%)	28.5–36.1	193 (23.5%)	20.7–26.4	<0.001
***Smoking status***								
Never	1398	582 (41.6%)	39.1–44.2	180 (31.3%)	27.6–35.2	402 (48.8%)	45.4–52.3	<0.001
Former		583 (41.7%)	39.1–44.3	301 (52.3%)	48.3–56.4	282 (34.3%)	31.1–37.6	
Current		233 (16.7%)	14.8–18.7	94 (16.3%)	13.5–19.5	139 (16.9%)	14.4–19.6	
***Pharmacotherapy***								
ACEi/ARB	1343	836 (62.2%)	59.6–64.8	368 (66.4%)	62.4–70.3	468 (59.3%)	55.9–62.7	0.010
Beta-blockers	1343	1237 (92.1%)	90.6–93.5	516 (93.1%)	90.8–95.0	721 (91.4%)	89.3–93.2	0.283
SGLT2 inhibitor	1343	876 (65.2%)	62.6–67.7	393 (70.9%)	67.1–74.6	483 (61.2%)	57.8–64.6	<0.001
MRA	1343	899 (66.9%)	64.4–69.4	397 (71.7%)	67.8–75.3	502 (63.6%)	60.2–66.9	0.003
ARNI	1410	325 (23.0%)	20.9–25.3	144 (24.9%)	21.5–28.5	181 (21.8%)	19.1–24.7	0.197
Oral anticoagulant	1343	716 (53.3%)	50.6–56.0	269 (48.6%)	44.4–52.7	447 (56.7%)	53.2–60.1	0.004
Antiplatelet	1343	565 (42.1%)	39.4–44.7	372 (67.1%)	63.2–71.0	193 (24.5%)	21.6–27.6	<0.001
Statins	1343	1048 (78.0%)	75.8–80.2	507 (91.5%)	89.0–93.6	541 (68.6%)	65.3–71.7	<0.001
***Patient-reported outcomes***								
KCCQ-12 score	1386	47.9 (28.7–73.4)	49.2–52.1	49.5 (31.3–74.0)	50.0–54.4	47.4 (27.6–72.4)	47.7–51.6	0.043
0–25		283 (20.4%)	18.4–22.6	100 (17.6%)	14.6–20.9	183 (22.4%)	19.6–25.3	0.173
25–50		440 (31.7%)	29.3–34.2	185 (32.6%)	28.8–36.5	255 (31.2%)	28.1–34.4	
50–75		337 (24.3%)	22.1–26.6	147 (25.9%)	22.4–29.6	190 (23.2%)	20.4–26.2	
75–100		326 (23.5%)	21.3–25.8	136 (23.9%)	20.6–27.6	190 (23.2%)	20.4–26.2	
***Clinical outcomes***								
All-cause mortality	1410	219 (15.5%)	13.7–17.5	90 (15.5%)	12.8–18.7	129 (15.5%)	13.2–18.1	>0.999
Follow-up, months	1410	14.8 (11.5–18.4)	14.5–15.1	15.0 (11.9–18.7)	14.6–15.5	14.5 (11.3–18.2)	14.2–15.0	0.189

Note. Data from the Heart Failure Observational Study (HEROES), a multicenter prospective registry across Polish cardiology centers. Twelve patients who died during index hospitalization were excluded prior to analysis. Continuous variables are reported as median (first quartile–third quartile). Categorical variables are reported as frequency (percentage). Moreover, 95% confidence intervals (CIs) were computed using the Jeffreys method for proportions and the Wilcoxon signed-rank method for medians. *p*-values were derived from the Wilcoxon rank-sum test for continuous variables and Pearson’s χ^2^ test for categorical variables. Bold *p*-values indicate statistical significance at α = 0.05. Abbreviations: ACEi—angiotensin-converting enzyme inhibitor; ARB—angiotensin receptor blocker; ARNI—angiotensin receptor neprilysin inhibitor; BP—blood pressure; HFmrEF—heart failure with mildly reduced ejection fraction; HFpEF—heart failure with preserved ejection fraction; HFrEF—heart failure with reduced ejection fraction; KCCQ-12—Kansas City Cardiomyopathy Questionnaire (12-item); MRA—mineralocorticoid receptor antagonist; NYHA—New York Heart Association; SGLT2—sodium glucose cotransporter 2; TIA—transient ischemic attack.

**Table 2:** Baseline characteristics stratified by sex within ischemic and non-ischemic heart failure groups.

Characteristic	Ischemic HF N = 579					Non-ischemic HF N = 831				
	Male N = 465	95% CI	Female N = 114	95% CI	*p*	Male N = 545	95% CI	Female N = 286	95% CI	*p*
***Demographics***										
Age, years	69.5 (63.4–76.2)	68.5–70.3	72.8 (66.9–77.6)	70.7–74.1	0.005	64.3 (52.6–72.4)	61.4–64.1	74.5 (67.4–81.7)	72.6–75.2	<0.001
***Anthropometrics and vital signs***										
BMI, kg/m^2^	27.7 (24.8–31.3)	27.7–28.6	27.1 (24.7–31.6)	27.0–29.1	0.692	28.4 (25.2–32.5)	28.5–29.5	27.8 (24.2–32.3)	27.6–29.1	0.069
Heart rate, bpm	75 (66–84)	74–77	75 (62–85)	73–80	0.962	80 (69–94)	80–83	79 (70–90)	78–83	0.790
Systolic BP, mmHg	126 (111–140)	125–128	127 (118–140)	125–132	0.223	128 (113–144)	126–130	129 (113–140)	125–130	0.825
Diastolic BP, mmHg	77 (70–83)	75–78	77 (70–82)	74–79	0.992	80 (70–89)	79–81	78 (67–82)	75–78	<0.001
***Heart failure classification***										
HFrEF	259 (64.8%)	60.0–69.3	46 (46.9%)	37.3–56.8	0.001	266 (56.4%)	51.9–60.8	56 (23.3%)	18.3–29.0	<0.001
HFmrEF	91 (22.8%)	18.8–27.0	27 (27.6%)	19.5–37.0		93 (19.7%)	16.3–23.5	51 (21.3%)	16.4–26.8	
HFpEF	50 (12.5%)	9.5–16.0	25 (25.5%)	17.7–34.8		113 (23.9%)	20.3–27.9	133 (55.4%)	49.1–61.6	
***Disease history***										
HF duration, years	4.6 (1.2–11.7)	5.7–7.3	4.2 (1.2–10.5)	4.2–7.3	0.420	2.2 (0.7–7.0)	3.2–4.3	2.3 (0.8–6.6)	3.0–4.3	0.637
< 1 year	90 (19.4%)	16.0–23.1	22 (19.3%)	12.9–27.3	0.979	184 (33.8%)	29.9–37.8	91 (31.8%)	26.6–37.4	0.797
1–3 years	106 (22.8%)	19.2–26.8	27 (23.7%)	16.6–32.1		124 (22.8%)	19.4–26.4	64 (22.4%)	17.8–27.5	
> 3 years	269 (57.8%)	53.3–62.3	65 (57.0%)	47.8–65.8		237 (43.5%)	39.4–47.7	131 (45.8%)	40.1–51.6	
Hospitalization	356 (76.6%)	72.6–80.2	90 (78.9%)	70.8–85.6	0.675	452 (82.9%)	79.6–85.9	233 (81.5%)	76.7–85.6	0.666
***Renal and heart function***										
eGFR, mL/min/1.73 m^2^	60.8 (46.7–77.1)	59.0–63.5	51.8 (35.3–71.8)	48.3–58.3	0.002	66.0 (52.5–81.4)	64.5–68.9	50.4 (36.1–67.1)	49.1–54.8	<0.001
LVEF, %	34.0 (25.0–43.0)	33.0–35.5	40.0 (30.2–49.5)	37.5–42.5	<0.001	35.0 (25.0–49.0)	35.0–37.5	50.0 (40.0–58.0)	47.5–51.0	<0.001
***Functional status***										
NYHA I–II	265 (58.2%)	53.7–62.7	54 (47.8%)	38.7–57.0	0.058	259 (49.1%)	44.9–53.4	111 (40.4%)	34.7–46.2	0.022
NYHA III–IV	190 (41.8%)	37.3–46.3	59 (52.2%)	43.0–61.3		268 (50.9%)	46.6–55.1	164 (59.6%)	53.8–65.3	
***Comorbidities***										
Arterial hypertension	334 (72.1%)	67.9–76.1	87 (77.7%)	69.3–84.6	0.285	320 (59.5%)	55.3–63.6	212 (74.4%)	69.1–79.2	<0.001
Atrial fibrillation	198 (42.8%)	38.3–47.3	57 (50.9%)	41.7–60.0	0.148	286 (53.2%)	48.9–57.4	182 (63.9%)	58.2–69.3	0.004
Diabetes mellitus	230 (49.7%)	45.1–54.2	51 (45.5%)	36.5–54.8	0.496	162 (30.1%)	26.3–34.1	89 (31.2%)	26.1–36.8	0.801
Stroke/TIA	43 (9.2%)	6.9–12.1	12 (10.5%)	5.9–17.1	0.811	36 (6.6%)	4.8–8.9	37 (12.9%)	9.4–17.2	0.003
MI	341 (73.7%)	69.5–77.5	82 (73.2%)	64.5–80.7	>0.999	39 (7.2%)	5.3–9.7	20 (7.0%)	4.5–10.4	>0.999
Stable angina	239 (51.6%)	47.1–56.1	61 (54.5%)	45.2–63.5	0.663	83 (15.4%)	12.6–18.7	47 (16.5%)	12.5–21.1	0.766
CKD	145 (31.3%)	27.2–35.6	40 (35.7%)	27.3–44.9	0.435	111 (20.6%)	17.4–24.2	82 (28.8%)	23.8–34.2	0.011
***Smoking status***										
Never	121 (26.1%)	22.3–30.3	59 (52.7%)	43.5–61.8	<0.001	199 (37.0%)	33.0–41.1	203 (71.2%)	65.8–76.2	<0.001
Former	261 (56.4%)	51.8–60.8	40 (35.7%)	27.3–44.9		223 (41.4%)	37.3–45.6	59 (20.7%)	16.3–25.7	
Current	81 (17.5%)	14.2–21.2	13 (11.6%)	6.7–18.5		116 (21.6%)	18.2–25.2	23 (8.1%)	5.3–11.7	
***Pharmacotherapy***										
ACEi/ARB	283 (64.0%)	59.5–68.4	85 (75.9%)	67.4–83.1	0.024	285 (54.8%)	50.5–59.0	183 (68.0%)	62.3–73.4	<0.001
Beta-blockers	412 (93.2%)	90.6–95.3	104 (92.9%)	87.0–96.6	>0.999	478 (91.9%)	89.3–94.0	243 (90.3%)	86.4–93.4	0.535
SGLT2 inhibitor	322 (72.9%)	68.6–76.8	71 (63.4%)	54.2–71.9	0.064	352 (67.7%)	63.6–71.6	131 (48.7%)	42.8–54.7	<0.001
MRA	322 (72.9%)	68.6–76.8	75 (67.0%)	57.9–75.2	0.264	370 (71.2%)	67.2–74.9	132 (49.1%)	43.1–55.0	<0.001
ARNI	127 (27.3%)	23.4–31.5	17 (14.9%)	9.3–22.3	0.009	157 (28.8%)	25.1–32.7	24 (8.4%)	5.6–12.0	<0.001
OAC	211 (47.7%)	43.1–52.4	58 (51.8%)	42.6–60.9	0.509	287 (55.2%)	50.9–59.4	160 (59.5%)	53.5–65.2	0.282
Antiplatelet	295 (66.7%)	62.3–71.0	77 (68.8%)	59.8–76.8	0.771	128 (24.6%)	21.1–28.5	65 (24.2%)	19.3–29.5	0.958
Statins	404 (91.4%)	88.5–93.7	103 (92.0%)	85.8–95.9	>0.999	362 (69.6%)	65.6–73.5	179 (66.5%)	60.8–72.0	0.423
***Patient-reported outcomes***										
KCCQ-12 score	53.7 (33.9–76.0)	51.8–56.8	40.6 (24.0–68.2)	38.3–48.7	<0.001	50.0 (30.2–76.0)	50.3–55.0	39.6 (21.9–64.1)	40.4–47.1	<0.001
0–25	71 (15.5%)	12.4–19.1	29 (26.1%)	18.6–34.8	0.003	103 (19.2%)	16.0–22.7	80 (28.5%)	23.4–33.9	0.003
25–50	143 (31.3%)	27.2–35.6	42 (37.8%)	29.2–47.1		163 (30.4%)	26.6–34.3	92 (32.7%)	27.5–38.4	
50–75	122 (26.7%)	22.8–30.9	25 (22.5%)	15.5–30.9		130 (24.2%)	20.7–28.0	60 (21.4%)	16.9–26.4	
75–100	121 (26.5%)	22.6–30.7	15 (13.5%)	8.1–20.8		141 (26.3%)	22.7–30.1	49 (17.4%)	13.3–22.2	
***Clinical outcomes***										
All-cause mortality	69 (14.8%)	11.8–18.3	21 (18.4%)	12.1–26.3	0.423	81 (14.9%)	12.1–18.0	48 (16.8%)	12.8–21.4	0.532
Follow-up, months	15.1 (11.9–18.9)	14.6–15.7	14.7 (11.7–17.7)	13.5–15.5	0.270	14.7 (11.7–18.4)	14.3–15.3	14.2 (10.7–17.9)	13.5–14.9	0.194

Note. Data from the Heart Failure Observational Study (HEROES). Continuous variables: median (Q1–Q3). Categorical variables: n (%). 95% CI computed via Jeffrey’s method (proportions) and Wilcoxon signed-rank method (medians). *p*-values from the Wilcoxon rank-sum test (continuous) and Pearson’s χ^2^ test (categorical), computed separately within each etiological stratum. Bold *p* < 0.05. Abbreviations: ACEi—angiotensin-converting enzyme inhibitor; ARB—angiotensin receptor blocker; ARNI—angiotensin receptor neprilysin inhibitor; BP—blood pressure; CKD—chronic kidney disease; HFmrEF/HFpEF/HFrEF—heart failure with mildly reduced/preserved/reduced ejection fraction; KCCQ-12—Kansas City Cardiomyopathy Questionnaire; MI—myocardial infarction; MRA—mineralocorticoid receptor antagonist; NYHA—New York Heart Association; OAC—oral anticoagulant; SGLT2—sodium glucose cotransporter 2; TIA—transient ischemic attack.

## Data Availability

Study URL: https://heroes.umed.pl. Database DOI: 10.60941/JVH1-5190.

## Supplementary Materials

The following supporting information can be downloaded at: https://www.mdpi.com/article/10.3390/jcm15124759/s1, Table S1: Summary of study design elements: exposure, outcome, estimand, and covariate framework; Table S2: Summary of entropy balancing weights and effective sample sizes by heart failure etiology; Table S3: Covariate balance diagnostics before and after entropy balancing for ischemic vs. non-ischemic heart failure comparison; Table S4: Adjusted hazard ratios from weighted cox proportional hazards regression models for the association between heart failure etiology (or sex) and all-cause mortality; Table S5: Sensitivity analyses: adjusted hazard ratios for the association between heart failure etiology and all-cause mortality under alternative propensity score weighting specifications; Figure S1: The study design, enrollment-to-analytical-stratification patient flow, and sequential methodological framework; Figure S2: Assessment of covariate balance before and after entropy balancing; Figure S3: Assessment of proportional hazards assumption; Figure S4: Subgroup-specific adjusted hazard ratios (non-ischemic vs. ischemic).

## References

[1] Savarese G., Becher P.M., Lund L.H., Seferovic P., Rosano G.M.C., Coats A.J.S. (2023). Global burden of heart failure: A comprehensive and updated review of epidemiology. Cardiovasc. Res..

[2] Groenewegen A., Rutten F.H., Mosterd A., Hoes A.W. (2020). Epidemiology of heart failure. Eur. J. Heart Fail..

[3] Tillman F., Kim J., Makhlouf T., Osae L. (2019). A comprehensive review of chronic heart failure pharmacotherapy treatment approaches in African Americans. Ther. Adv. Cardiovasc. Dis..

[4] Elendu C., Amaechi D.C., Elendu T.C. (2023). Heart failure and diabetes: Understanding the bidirectional relationship. Medicine.

[5] Núñez J., Miñana G., Bodí V., Sanchis J., Núñez E., Husser O., Chorro F.J., Llàcer A. (2015). Ischemic etiology and prognosis in men and women with acute heart failure. Int. J. Cardiol..

[6] Pecini R., Møller D.V., Torp-Pedersen C., Hassager C., Køber L. (2011). Heart failure etiology impacts survival of patients with heart failure. Int. J. Cardiol..

[7] Balmforth C., Simpson J., Shen L., Jhund P.S., Lefkowitz M., Rizkala A.R., Rouleau J.L., Shi V., Solomon S.D., Swedberg K. (2019). Outcomes and effect of treatment according to etiology in HFrEF: An analysis of PARADIGM-HF. JACC Heart Fail..

[8] French J.K., Armstrong P.W., Cohen E., Kleiman N.S., O’Connor C.M., Hellkamp A.S., Stebbins A., Holmes D.R., Hochman J.S., Granger C.B. (2011). Cardiogenic shock and heart failure post-percutaneous coronary intervention in ST-elevation myocardial infarction: Observations from the Assessment of Pexelizumab in Acute Myocardial Infarction. Am. Heart J..

[9] Villaschi A., Pagnesi M., Stolfo D., Sinagra G., Metra M., Cleland J.G.F., Savarese G. (2023). Ischemic etiology in advanced heart failure: Insight from the HELP-HF registry. Am. J. Cardiol..

[10] Drożdż J., Morawiec R., Drozd M. (2025). Rationale, objectives and design of the HEart failuRe ObsErvational Study of the Polish Cardiac Society (HEROES). Kardiol. Pol..

[11] Udelson J.E. (2019). Is heart failure etiology destiny? Outcome and therapeutic implications. JACC Heart Fail..

[12] Vergaro G., Ghionzoli N., Innocenti L. (2019). Noncardiac versus cardiac mortality in heart failure across EF spectrum. J. Am. Heart Assoc..

[13] Kozman K., Ferrannini G., Benson L. (2025). Etiology of heart failure across the ejection fraction spectrum and association with prognosis. JACC Heart Fail..

[14] Doryńska A., Drohomirecka A., Topór-Mądry R., Łazarczyk H., Zieliński T., Rywik T.M. (2025). Changes in the prevalence of heart failure phenotypes over time and their association with patient prognosis. Pol. Arch. Intern. Med..

[15] Walli-Attaei M., Joseph P., Johansson I. (2024). Characteristics, management, and outcomes in women and men with congestive heart failure in 40 countries at different economic levels: An analysis from the G-CHF registry. Lancet Glob. Health.

[16] Lam C.S.P., Arnott C., Beale A.L. (2019). Sex differences in heart failure. Eur. Heart J..

[17] Vedin O., Lam C.S.P., Koh A.S. (2017). Significance of ischemic heart disease in heart failure across EF spectrum. Circ. Heart Fail..

[18] Spitaleri G., Zamora E., Cediel G., Codina P., Santiago-Vacas E., Domingo M., Lupón J., Santesmases J., Diez-Quevedo C., Troya M.I. (2022). Cause of death in heart failure based on etiology: Long-term cohort study of all-cause and cardiovascular mortality. J. Clin. Med..

[19] Rywik T.M., Topór-Mądry R., Drohomirecka A. (2025). Is etiology the dominant modifier of prognosis in HF phenotypes. Kardiol. Pol..

[20] Felker G.M., Shaw L.K., O’Connor C.M. (2002). A standardized definition of ischemic cardiomyopathy for use in clinical research. J. Am. Coll. Cardiol..

[21] Deswal A., Bozkurt B. (2006). Comparison of morbidity in women versus men with heart failure and preserved ejection fraction. Am. J. Cardiol..

[22] Regitz-Zagrosek V., Oertelt-Prigione S., Prescott E., Franconi F., Gerdts E., Foryst-Ludwig A., Maas A.H.E.M., Kautzky-Willer A., Knappe-Wegner D., Kintscher U. (2016). Gender in cardiovascular diseases: Impact on clinical manifestations, management, and outcomes. Eur. Heart J..

[23] Martínez-Sellés M., García Robles J.A., Prieto L., Domínguez Muñoa M., Frades E., Díaz-Castro O., Almendral J. (2003). Systolic dysfunction is a predictor of long-term mortality in men but not in women with heart failure. Eur. Heart J..

[24] Ghali J.K., Krause-Steinrauf H.J., Adams K.F., Khan S.S., Rosenberg Y.D., Yancy C.W., Young J.B., Goldman S., Peberdy M.A., Lindenfeld J. (2003). Gender differences in advanced heart failure: Insights from the BEST study. J. Am. Coll. Cardiol..

[25] Dunlay S.M., Roger V.L. (2012). Gender differences in the pathophysiology, clinical presentation, and outcomes of ischemic heart failure. Curr. Heart Fail. Rep..

[26] Frazier C.G., Alexander K.P., Newby L.K. (2007). Associations of gender and etiology with outcomes in heart failure with systolic dysfunction: A pooled analysis of five randomized controlled trials. J. Am. Coll. Cardiol..

[27] Martínez-Sellés M., Doughty R.N., Poppe K. (2012). Gender and survival in patients with heart failure: Interactions with diabetes and etiology. Results from the MAGGIC individual patient meta-analysis. Eur. J. Heart Fail..

[28] Schall M.B., Flannery J. (2004). Undertreatment of women with heart failure: A reversible outcome on hospital readmission. Lippincotts Case Manag..

