# Supplementary material for: Association Between Heart Failure Etiology and All-Cause Mortality with Sex-Specific Considerations: Insights from the HEROES Registry
Source: J Clin Med. 2026 Jun 18;15(12):4759. doi: 10.3390/jcm15124759 (PMC13302107; doi:10.3390/jcm15124759)
Supplement: Supplementary file 1 [file jcm-15-04759-s001.zip › jcm-4328455-supplementary tables.pdf]

Table S1. Summary of study design elements: exposure, outcome, estimand, and covariate framework.

Element	Specification
**Study design**	Prospective, multicentre, observational cohort (HEROES registry)
**Enrolment**	N = 1,422 consenting patients; 12 in-hospital deaths excluded → N = 1,410
**Exposure**	Etiological classification: ischemic (n = 579) vs. non-ischemic (n = 831), treated as a binary variable
**Non-ischaemic subcategories**	Dilated cardiomyopathy (n = 191), hypertensive HF (n = 170), valvular HF (n = 151), other (n = 319)
**Primary outcome**	All-cause mortality (time-to-event), follow-up in months from index enrolment
**Censoring**	Administrative censoring at end of observation or last known contact date
**Target estimand**	Average treatment effect (ATE) estimated via inverse probability of treatment weighting
**Effect measure**	Adjusted hazard ratio (aHR) with 95% CI from weighted Cox PH model with robust SE
**PSW method**	Entropy balancing (Hainmueller, 2012); exact first-moment balance; ATE estimand
**PSW covariates (13)**	Age, sex, BMI, heart rate, systolic BP, diastolic BP, HF duration, type of visit, NYHA class, diabetes mellitus, CKD, atrial fibrillation, arterial hypertension
**Excluded from PSW**	MI, stable angina (definitional); antiplatelet, statins, SGLT2i, MRA, OAC, ACEi/ARB, ARNI (post-exposure mediators/treatments)
**Balance threshold**	$\lvert SMD \rvert < 0.10$ (Austin, 2009)
**Sex-stratified analyses**	Separate PSW models within ischaemic and non-ischaemic subgroups (Figures 2–3); 12 covariates (sex excluded as exposure)
**Phenotype-stratified**	Separate PSW within HFrEF, HFmrEF, HFpEF (Figure 4); 13 covariates

Note. PSW—propensity score weighting; SMD—standardised mean difference; SE—standard error; PH—proportional hazards; CKD—chronic kidney disease; MI—myocardial infarction; BP —blood pressure; BMI —body mass index.

Table S2. Summary of entropy balancing weights and effective sample sizes by heart failure etiology.

Group	Weight range	CV	MAD	ESS (raw)	ESS (weighted)
Ischemic	0.18—4.34	0.55	0.39	565	435.7
Non-ischemic	0.34—3.27	0.36	0.28	797	705.1

Note. Propensity score weights estimated via entropy balancing (Hainmueller, 2012) targeting the average treatment effect (ATE). ESS (effective sample size) quantifies information retained after weighting, accounting for variance inflation: $ESS = (\sum w_i)^2 / \sum w_i^2$. CV—coefficient of variation of weights. MAD—mean absolute deviation. Weight ranges reflect the minimum and maximum individual weights across all patients within each group.

Table S3. Covariate balance diagnostics before and after entropy balancing for ischemic vs. non-ischemic heart failure comparison.

Covariate	SMD (unadjusted)	SMD (adjusted)	Balance status
Age, years	-0.29	0.00	Balanced
Sex (female)	0.34	0.00	Balanced
Body mass index, kg/m²	-0.16	0.00	Balanced
Heart rate, bpm	0.29	0.00	Balanced
Systolic BP, mmHg	0.06	0.00	Balanced
Diastolic BP, mmHg	0.18	0.00	Balanced
HF duration, years	-0.27	0.00	Balanced
Visit type (outpatient)	-0.12	0.00	Balanced
NYHA III–IV	0.20	0.00	Balanced
Diabetes mellitus	-0.39	0.00	Balanced
Chronic kidney disease	-0.20	0.00	Balanced
Atrial fibrillation	0.24	0.00	Balanced
Arterial hypertension	-0.18	0.00	Balanced

Note. SMD—standardised mean difference, computed using pooled standard deviations as denominators (Austin, 2009). Positive SMD indicates higher values in the non-ischemic group; negative SMD indicates higher values in the ischemic group. Balance threshold: $|SMD| < 0.10$, in accordance with conventional guidelines for minimising residual confounding. Post-weighting SMDs of exactly 0.0000 confirm perfect first-moment balance achieved by entropy balancing. All 13 covariates met the pre-specified balance criterion (13/13 balanced).

Table S4. Adjusted hazard ratios from weighted cox proportional hazards regression models for the association between heart failure etiology (or sex) and all-cause mortality.

Model	N	Events	aHR	95% CI	P
**Model 1:** Etiology, all patients	1,362	207	1.16	0.85—1.58	0.363
**Model 2:** Sex, ischemic HF	565	84	1.14	0.67—1.94	0.633
**Model 3:** Sex, non-ischemic HF	797	123	0.85	0.56—1.27	0.420
**Model 4:** Doubly robust, all patients	1,362	207	1.12	0.82—1.54	0.469

Note. aHR—adjusted hazard ratio from weighted Cox proportional hazards regression with robust (sandwich) standard errors. Models 1–3: PSW weights from entropy balancing (ATE estimand); single-predictor models (confounding addressed via weighting). Model 4: doubly robust specification incorporating both PSW weights and direct covariate adjustment (age, sex, NYHA class, diabetes mellitus, chronic kidney disease). Model 1 reference: ischemic aetiology. Models 2–3 reference: male sex. Models 2 and 3 employ separate PSW models fitted within each aetiological subgroup.

Table S5. Sensitivity analyses: adjusted hazard ratios for the association between heart failure etiology and all-cause mortality under alternative propensity score weighting specifications.

Weighting method	aHR	95% CI	P
**Entropy balancing (primary)**	1.16	0.85—1.58	0.363
Generalised boosted models (GBM)	1.09	0.82—1.46	0.542
Covariate balancing PS (CBPS)	1.05	0.77—1.42	0.773
IPTW, logistic regression (99th %ile trimmed)	1.06	0.78—1.44	0.697
**Entropy balancing + HFC (N = 1,210)**	1.17	0.84—1.64	0.354

Note. All models compare non-ischemic vs. ischemic aetiology (reference category) with robust (sandwich) standard errors. The primary analysis uses entropy balancing (Hainmueller, 2012) with 13 covariates. GBM—gradient boosting with optimisation for mean effect size balance (n.trees = 5,000). CBPS—covariate balancing propensity scores, jointly optimising balance and prediction (Imai & Ratkovic, 2014). IPTW—inverse probability of treatment weighting via logistic regression, with weights trimmed at the 99th percentile to mitigate extreme values. HFCheart failure classification (HFrEF/HFmrEF/HFpEF) added as a 14th covariate; sample reduced from N = 1,362 to N = 1,210 due to 14.4% missing LVEF data. Concordance across all five specifications (aHR range: 1.05–1.17) demonstrates robustness of the primary finding.
