# Supplementary figures and images for: Association Between Heart Failure Etiology and All-Cause Mortality with Sex-Specific Considerations: Insights from the HEROES Registry
Source: J Clin Med. 2026 Jun 18;15(12):4759. doi: 10.3390/jcm15124759 (PMC13302107; doi:10.3390/jcm15124759)

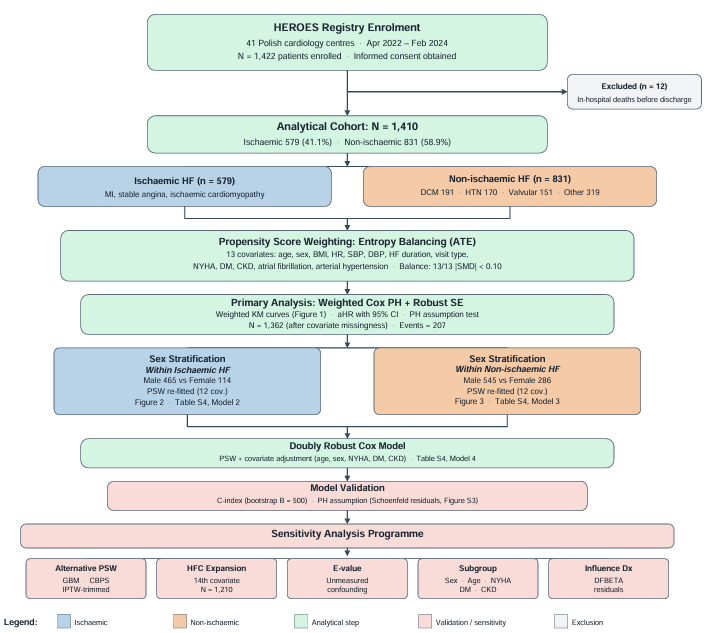

Supplement: Supplementary file 1 [file jcm-15-04759-s001.zip › Figure S1.png]

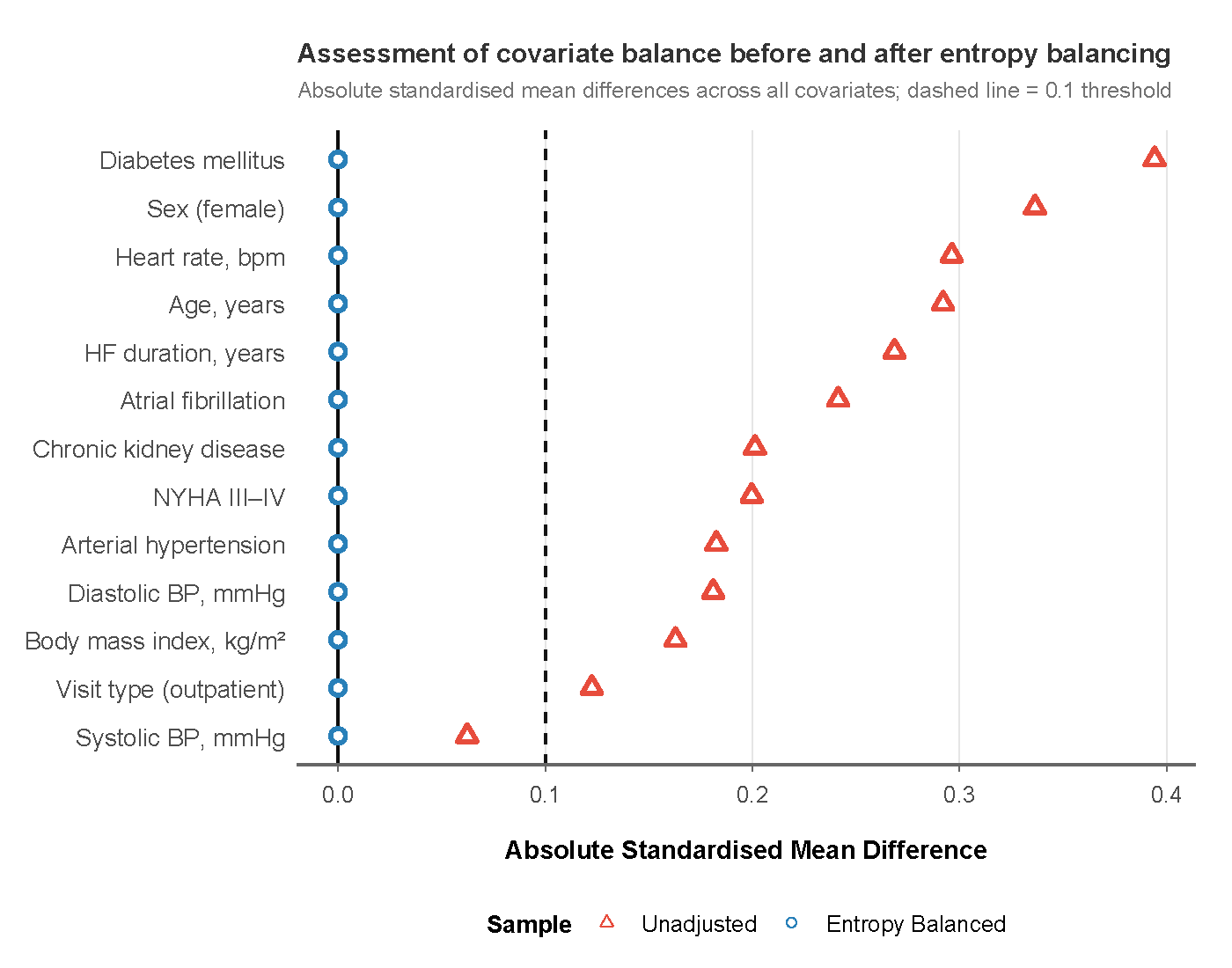

Supplement: Supplementary file 1 [file jcm-15-04759-s001.zip › Figure S2.png]

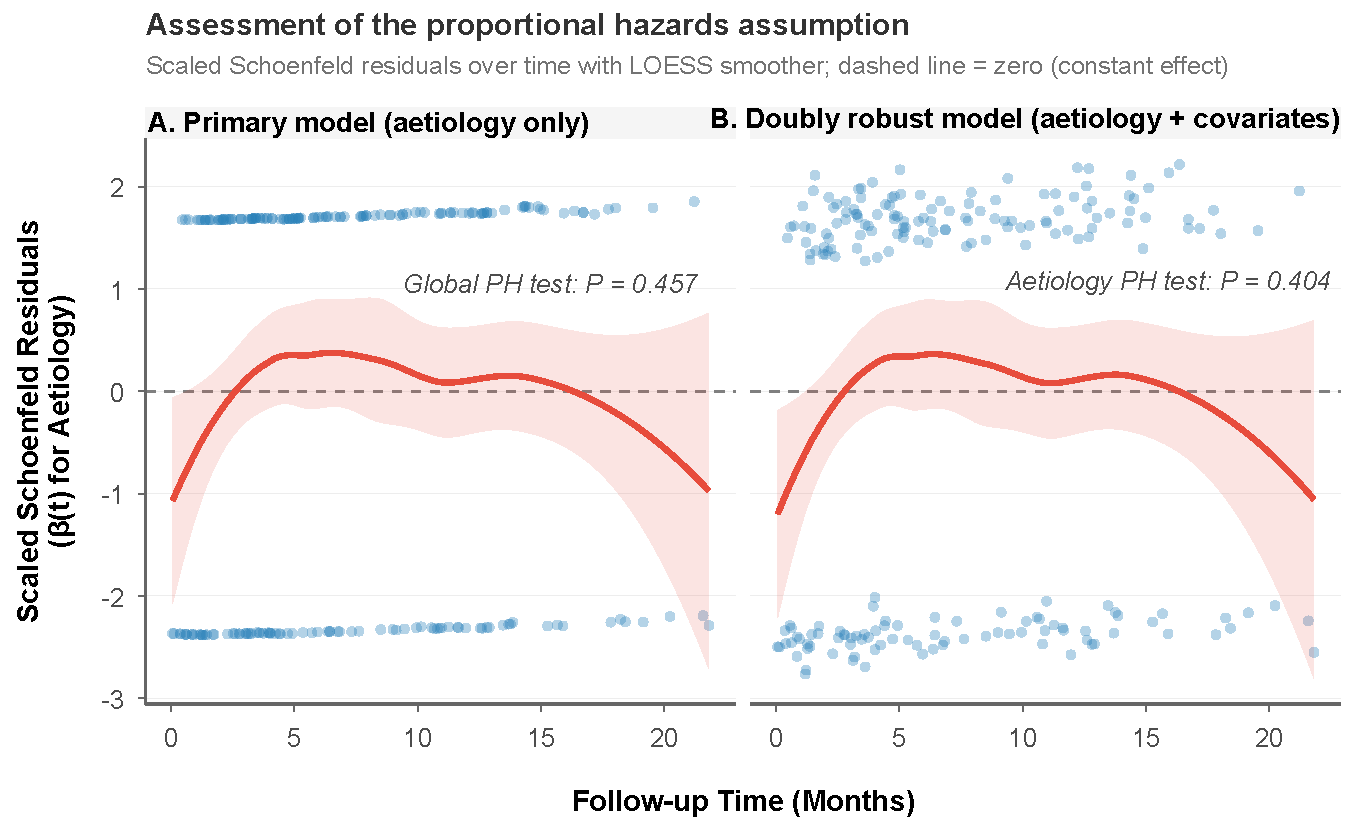

Supplement: Supplementary file 1 [file jcm-15-04759-s001.zip › Figure S3.png]

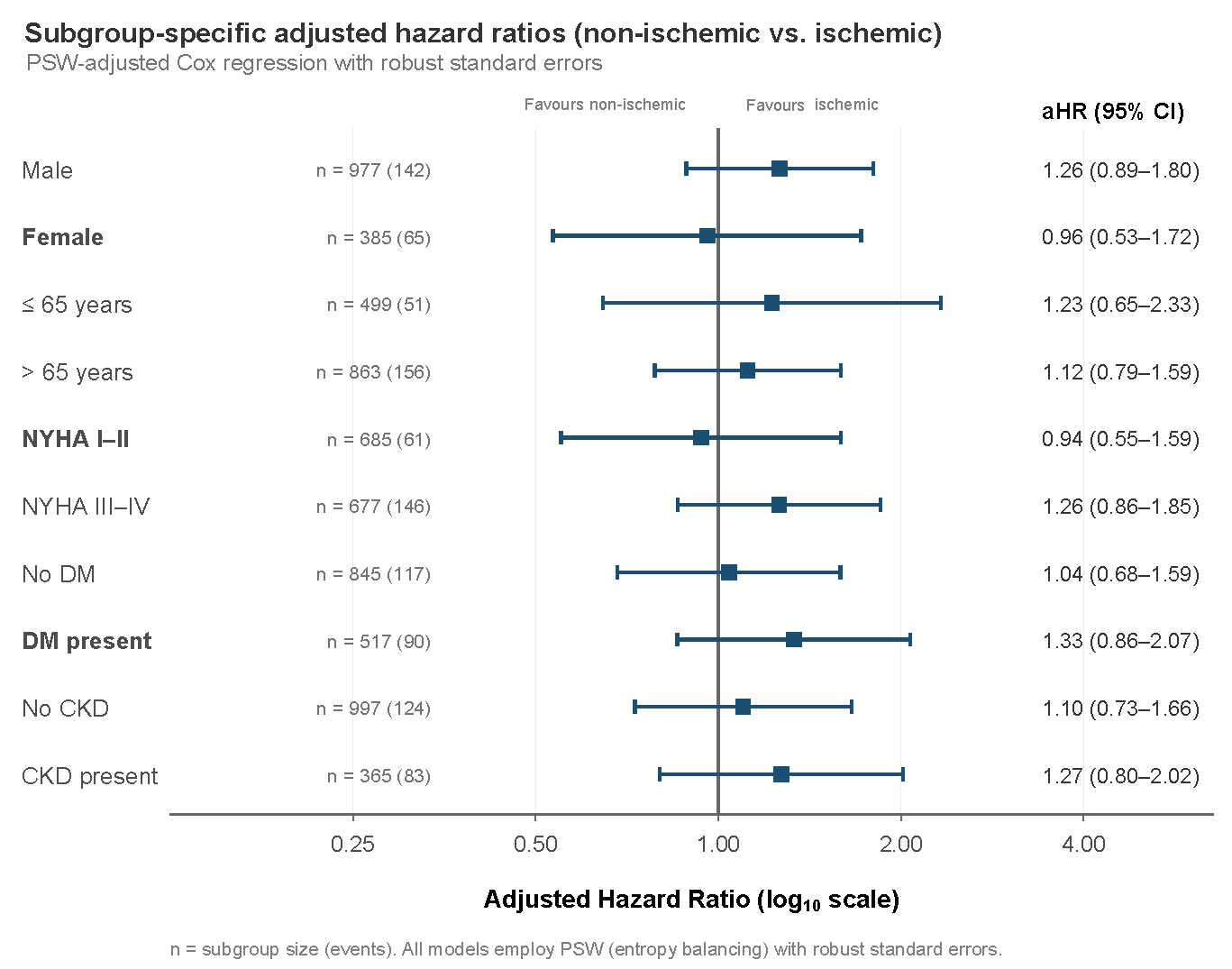

Supplement: Supplementary file 1 [file jcm-15-04759-s001.zip › Figure S4.png]
